# Acute kidney injury in hospitalized patients with real-life analysis of incidence and clinical impact in Italian hospitals (the SIN-AKI study)

**DOI:** 10.1038/s41598-025-96236-8

**Published:** 2025-04-24

**Authors:** Pasquale Esposito, Francesca Cappadona, Stefania Prenna, Marita Marengo, Marco Fiorentino, Paolo Fabbrini, Alessandro Domenico Quercia, Erika Naso, Francesco Garzotto, Elisa Russo, Valentina Zanetti, Riccardo Piscia, Andrea Capponi, Giuseppe Castellano, Vincenzo Cantaluppi

**Affiliations:** 1 Department of Internal Medicine and and Medical Specialties (DIMI), University of Genova, Genova, Italy; 2 Unit of Nephrology, Dialysis and Transplantation, IRCCS Ospedale Policlinico San Martino, Genova, Italy; 3 Nephrology and Kidney Transplantation Unit, Department of Translational Medicine (DIMET), University of Piemonte Orientale (UPO), AOU Maggiore Della Carità, Novara, Italy; 4 Nephrology and Dialysis Unit, Aslcn1, Cuneo, Italy; 5 Nephrology, Dialysis and Transplantation Unit, Department of Precision and Regenerative Medicine and Ionian Area, University of Bari Aldo Moro, Bari, Italy; 6 Nephrology and Dialysis Unit, ASST Nord Milano, Milano, Italy; 7 Department of Cardiac Thoracic Vascular Sciences and Public Health, Unit of Biostatistics, Epidemiology and Public Health, University of Padua, Padua, Italy; 8 Department of Nephrology, Dialysis and Renal Transplantation, Fondazione IRCCS Ca’ Granda Ospedale Maggiore Policlinico, Milan, Italy; 9 The AKI and Extracorporeal Blood Purification Therapies Project Group, Italian Society of Nephrology (SIN), Rome, Italy

**Keywords:** Acute kidney injury, Hospitalization, Mortality, Renal recovery, Outcomes, Acute kidney injury, Health policy

## Abstract

Acute Kidney Injury (AKI) is a common condition with significant impact on morbidity, mortality, and healthcare costs. This study explores the epidemiology of AKI, highlighting key factors and outcomes. In a retrospective study we evaluated patients admitted to hospital from 2016 to 2019, excluding those with pre-existing chronic kidney disease (CKD) stages 4–5. Data were extracted from hospital databases, with AKI defined by changes in serum creatinine (sCr) according to KDIGO criteria. Additionally, AKI was classified as “de novo” or as AKI on CKD in the subgroup of patients with available pre-hospital eGFR. Outcomes included mortality, hospital stay duration (LOS), AKI recovery, and persistent AKI. Of 87,087 patients, 17,946 (20.6%) developed AKI. AKI patients were older, with more comorbidities, and had significantly higher mortality (17.7% vs. 4.3%, p < 0.001). AKI was associated with in-hospital mortality (HR 1.23, 95% CI 1.16–1.30), longer LOS, and ICU admission. Mortality increased with AKI severity. Considering the 34,285 patients (39% of the total cohort) with pre-hospital eGFR, AKI occurred in 17.3% patients without previous CKD and in 31.1% of patients with previous CKD. These patients presented higher incidence of ICU admission and mortality. Additionally, 17.6% of AKI patients had persistent kidney dysfunction at discharge, often requiring extended hospitalization and ICU care. The substantial impact of AKI on both short- and potentially long-term health emphasizes the importance of early detection, personalized management, and structured follow-up to enhance outcomes and reduce CKD progression risk.

## Introduction

Acute Kidney Injury (AKI), one of the most frequent and probably underestimated syndromes in hospitalized patients, is commonly defined as an abrupt decline of renal function evaluated by the increase of serum creatinine levels and by the concomitant decrease of urinary output categorized according to the KDIGO 2012 criteria^1^. Previous studies clearly showed that the most severe forms of AKI (stages 2–3 KDIGO) are associated with increased risk of mortality, length of hospital stay and healthcare costs mainly due to frequent re-hospitalizations and development of comorbidities^2^. Moreover, AKI represents an independent risk factor for the progression toward chronic kidney disease (CKD)^3,4^. AKI has recently emerged as a major public health concern with high human and financial costs. In the US, AKI leads to an increase of the hospitalization costs that ranges $5.4 to $24 billion: the higher costs are related to the need of renal replacement therapies (RRT) leading to a longer hospital stay^5^.

Moreover, we have recently found a significant discrepancy in AKI epidemiology depending on the definition used. Specifically, reliance solely on administrative data, as opposed to diagnosis based on serum creatinine changes, substantially underestimates AKI incidence in hospitalized patients^6^.

Given these challenges, there is an urgent need for new studies to accurately assess the actual incidence of AKI. This would provide hospitals and policymakers with the necessary data to develop integrated healthcare strategies aimed at reducing AKI-related costs.

This is particularly relevant for elderly patients who are more prone to develop AKI in consideration of the loss of renal functional reserve due to aging and the presence of different chronic comorbidities such as diabetes, hypertension and heart failure^7^. In a European hospitalized cohort, the average age of AKI patients was 76 years^8^. Moreover, it has been shown that age-related yearly incidence of AKI increased from 17 per million in adults under age 50 to 949 per million in those aged 80–89^9^.

Most of the published studies on AKI referred to critically ill patients admitted to Intensive Care Units (ICU) in which the main driver of renal dysfunction is sepsis^10^. However, recent reports showed that AKI is a frequent complication also in low- and medium-intensity care settings, representing the first cause of Nephrology consultancy worldwide^11–13^.

To date, we still have some unmet needs in the management of AKI in hospitalized patients: first, an earlier diagnosis of AKI may limit the worsening of the syndrome with the need of RRT, decreasing the length of hospital stay, thus improving outcomes. Secondly, the identification of AKI patients more prone to the progression toward CKD at hospital discharge remains challenging both for clinical and organizational causes^14^. AKI patients should enter a program of specialized follow-up aimed to assess renal function and the development of distant organ complications^15,16^.

This study aimed to assess in-hospital incidence of AKI using a standardized epidemiologic model, paving the way for improved AKI recognition, specialized post-discharge care, and biomarker discovery to better understand the progression from AKI to CKD.

## Materials and methods

### Study design and patient enrolment

We performed a retrospective observational study in patients aged ≥ 18 years hospitalized at the Policlinico Universitario “San Martino”, Genova (Italy) and at the Azienda Ospedaliera Universitaria “Maggiore della Carità”, Novara (Italy), from January 1, 2016, to December 31, 2019. Patients were included at the time of first hospital admission. All subjects with chronic kidney disease (CKD) stage 4 and 5 identified by the ICD- 9-CM (International Classification of Disease, 9 th Revision, Clinical Modification) diagnosis codes reported on the Hospital Discharge Form (HDF) and/or in regular outpatient clinic pre-dialysis follow-up, were not included in the research algorithm. Both institutional review boards approved the study protocol (Genova: N. Registro CER Liguria: 515/2020; Novara: Protocollo 530/CE, Studio n. CE 220/19 NOV-AKI Study) and waived the need for informed consent. The study was performed in accordance with the Declaration of Helsinki.

### Data collection and definitions

All data were obtained from the hospital electronic database. We exported the following demographic, clinical and laboratory data: age, sex, comorbidities, serum creatinine (sCr), admission ward, length of hospital stay (LOS), death, and outcome. The most frequent comorbidities were identified by using ICD- 9-CM codes^17^. The values of sCr were collected at different time points: hospital admission, hospital discharge, peak and nadir levels during hospitalization. Only patients with at least two sCr determinations were admitted to the study.

For the primary objectives of the study, the lowest sCr value recorded during hospitalization was assumed as the baseline for all included patients^18^.

AKI incidence was then determined based on changes in sCr, calculated as the ratio of peak sCr to the lowest sCr during hospitalization (peak sCr/lowest sCr).

We defined and graded AKI according to the Kidney Disease Improving Global Outcomes (KDIGO) Clinical Practice Guideline, based only on sCr changes. In accordance with KDIGO, stage 1, 2 or 3 AKI was defined as sCr increase of 1.5 to 1.9 times, 2 to 2.9 times, and ≥ 3 times (or need of dialysis) in respect to baseline sCr level respectively^1^. Urine output was not considered due to the retrospective nature of the study and to the limited collected data outside the Intensive Care Unit (ICU).

Notably, based on the available data, we were unable to determine which patients required dialysis during hospitalization. However, it is likely that these patients, considering their low baseline creatinine levels—potentially influenced by dialysis—were classified within the AKI stage 3 group.

When available in the laboratory database, we also collected sCr and estimated Glomerular Filtration Rate (eGFR by using CKD-EPI Formula) performed from day 7 to 180 before hospitalization to identify pre-existing CKD defined as eGFR < 60 ml/min/1.73 m^2^. Then, in the subgroup of patients with available pre-hospital eGFR, we differentiated and compared patients with “de novo” AKI defined as an AKI episode in absence of pre-existing CKD, and AKI on CKD defined as AKI that developed in patients with pre-existing CKD.

AKI persistence or recovery were defined by calculating the ratio between sCr at discharge and the lowest sCr during hospitalization (discharge sCr/lowest sCr): since the inclusion in the KDIGO criteria requires a discharge sCr/lowest sCr ≥ 1.5, we considered patients with persistent AKI accordingly. Conversely, patients with a ratio < 1.5 were classified as recovery AKI.

### Outcomes

The following outcomes were considered: a) incident in-hospital AKI; b) overall mortality; c) length of hospital stay (LOS); d) type of hospital discharge (protected vs. at home); e) persistence or recovery AKI at hospital discharge.

### Statistical analysis

Normally distributed variables are presented as mean ± 1SD and compared using an independent or paired t-test as appropriate. Logarithmically transformed values of skewed variables were used for the statistical analysis. Comparisons between groups were made by analysis of variance. Comparisons of proportions were made using the χ2-test or Fisher’s exact test when appropriate. The incidence rate of AKI was calculated. Univariate and multivariate logistic regression analyses were used to describe the relationship between all available clinical variables of biological relevance and the presence of AKI. Odds ratios and 95% confidence intervals were calculated by exponentiation of logistic regression coefficients. Time-to-event analyses were performed using: (i) the Kaplan–Meier method for survival curves estimation and log-rank test to compare them; (ii) univariate and multivariate Cox regression models: risk was reported as hazard ratios (HR) along with their 95% confidence intervals (CI). Covariates included all available clinical variables with biological plausibility. The time variable was defined as the interval time between the baseline date and the date of endpoint occurrence or the last available follow-up. Power analysis showed that the number of individuals in the database (n = 87,087) represented a sample largely sufficient to avoid b error also after stratification by AKI. Statistical calculations were performed by STATA package, version 14.2 (StataCorp, 4905 Lakeway Drive, College Station, Texas 77,845 USA). The null hypothesis was rejected for values of p < 0.05.

## Results

### Patients’ characteristics

We collected data from 87,087 hospitalized patients with a mean age of 69.2 ± 17.7 years, 43,467 (49.9%) males. As reported in the HDF, 8455 (9.7%) patients were diabetic, 7767 (8.9%) had heart failure (HF) and 5924 (6.8%) had pre-existing CKD. Sepsis occurred in 3361 (3.9%) cases. At hospital admission, mean sCr was 1.12 ± 0.98 mg/dl, corresponding to an eGFR of 90.1 ± 16 ml/min. Most of the patients were admitted to low or medium intensity care settings, whereas 3147 (3.6%) to ICU (Table 1).

**Table 1:** Main clinical characteristics of hospitalized patients according to AKI development.

	All	No-AKI	AKI	P (No-AKI vs AKI)
N,	87,087	69,141 (79.4)	17,946 (20.6)	
Age, years	69.2 ± 17.7	66.7 ± 18.1	74.8 ± 14.7	< 0.001
Sex, male%	43,467 (49.9)	34,869 (50.4)	8598 (47.9)	< 0.001
Comorbidities, %				
Diabetes	8455 (9.7)	6446 (9.3)	2009 (11.2)	< 0.001
Heart failure	7767 (8.9)	5122 (7.4)	2645 (14.7)	< 0.001
Sepsis	3361 (3.9)	1448 (2.1)	1913 (10.7)	< 0.001
CKD	5924 (6.8)	4220 (6.1)	1704 (9)	< 0.001
sCr at admission (mg/dl)	1.12 ± 0.98	1 ± 1	1.55 ± 1.53	< 0.001
eGFR at admission (ml/min)	90.1 ± 16	91 ± 15	81.6 ± 16	< 0.001
				AKI incidence (%)*
Hospital Ward				
Medicine	37,902 (43.5)	30,951 (44.7)	6951 (38.7)	10.4
Surgery	22,569 (25.9)	18,949 (27.4)	3620 (20.2)	16
ICU	3147 (3.6)	1666 (2.4)	1481 (8.2)	47
Emergency Medicine	23,467 (26.9)	17,574 (25.4)	5893 (32.8)	25.1

Abbreviations:* CKD*: Chronic Kidney Disease, *sCr*: Serum Creatinine, *ICU*: Intensive Care Unit, *AKI*: Acute Kidney Injury.

### Incidence and risk factors for AKI

AKI developed in 17,946 (20.6%) patients. In respect to the No-AKI population, patients with AKI were significantly older and with a higher prevalence of females. Moreover, in respect to the No-AKI group, AKI patients presented a higher prevalence of all the recorded comorbidities, including CKD (9 vs 6.1% of the No-AKI group, p < 0.001), diabetes (11.2 vs 9.3%, p < 0.001), heart failure (14.7 vs 7.4%, p < 0.001) and sepsis (10.7 vs 2.1%, p < 0.001) (Table 1).

As expected, analyzing admission data, we found that patients experiencing AKI showed higher sCr levels (1.55 ± 1.53 vs 1 ± 1 mg/dl, p < 0.001) and lower eGFR (81.6 ± 16 vs 91 ± 15 ml/min, p < 0.001) when compared with the No-AKI group. AKI incidence was significantly higher in patients admitted to ICU (47%) and emergency wards (25%) compared with general internal medicine and surgery units (10.4% and 16%, respectively).

We then analyzed the clinical determinants of AKI by univariate and multivariate logistic regression analyses (Table 2). For the latter, two independent models were generated since the variables *CKD* reported in the HDF and *admission sCr* were found to be correlated during preliminary analysis. The results of logistic regressions demonstrated that in both univariate and multivariate analyses, demographic characteristics (age, female sex), length of hospital stay, ICU admission, main comorbidities (except for diabetes), and admission sCr were significantly associated with the risk of AKI development (Table 2).

**Table 2:** Logistic models for AKI development.

Risk factors	Univariate			Multivariate model 1			Multivariate model 2		
	OR	95% CI	p	OR	95% CI	p	OR	95% CI	p
Sex (female)	1.11	1.07–1.14	< 0.0001	1.08	1.04–1.12	< 0.0001	1.23	1.18–1.27	< 0.0001
Age	1.03	1.02–1.03	< 0.0001	1.03	1.02–1.03	< 0.0001	1.02	1.02–1.02	< 0.0001
Comorbidities									
Diabetes	1.23	1.16–1.29	< 0.0001	1.05	0.99–1.11	0.08	1.02	0.96–1.08	0.58
HF	2.16	2.05–2.27	< 0.0001	1.51	1.43–1.6	< 0.0001	1.43	1.35–1.51	< 0.0001
Sepsis	5.58	5.2–5.9	< 0.0001	3.61	3.35–3.9	< 0.0001	3.2	2.96–3.4	< 0.0001
CKD	1.61	1.52–1.71	< 0.0001	1.38	1.28–1.47	< 0.0001	-	-	**-**
Admission sCr	1.67	1.64–1.70	< 0.0001	-	-	-	1.55	1.53 − 1.58	< 0.0001
Medical Ward	Ref								
ICU stay	4.38	4.11–4.67	< 0.0001	4.65	4.33–4.9	< 0.0001	4.73	4.4–5.1	< 0.0001
LOS (days)	1.21	1.17–1.36	< 0.0001	1.05	1.05–1.06	< 0.0001	1.05	1.05–1.06	< 0.0001

Abbreviations: OR odds ratio, CI confidence interval, HF heart failure, CKD chronic kidney disease, sCr serum creatinine, ICU intensive care unit, LOS length of stay.

### Correlation of outcomes with AKI development

The in-hospital mortality rate was significantly higher in the AKI group compared to the No-AKI group (Table 3). Additionally, we observed that a higher number of AKI patients were admitted to ICU compared to low- and medium-intensity care settings. These patients also experienced a significantly longer LOS compared to No-AKI patients. At hospital discharge, AKI patients still exhibited elevated sCr levels and required a higher number of protected follow-up to ensure the continuity of care.

**Table 3:** Clinical outcomes according to AKI development.

	All	No-AKI	AKI	P (No-AKI vs AKI)
	87,087	69,141	17,946	
In-hospital outcomes				
Mortality rate, %	6156 (7.07)	2984 (4.3)	3172 (17.7)	< 0.0001
ICU admission, %	3147 (3.6)	1666 (2.4)	1481 (8.2)	< 0.0001
LOS (days)	11.1 ± 13	8.9 ± 11.12	19.2 ± 16	< 0.0001
Discharge status- alive				
Discharge at home, %	63,666 (73.1)	54,284 (78.5)	8382 (52.8)	< 0.0001
Protected discharge, %	17,265 (19.8)	11,873 (17.7)	5392 (30.05)	< 0.0001
sCr (mg/dl)	1.05 ± 1	1.00 ± 0.7	1.27 ± 1.1	< 0.0001
eGFR ml/min	90.89 ± 16	92.46 ± 15.7	84.86 ± 15.9	< 0.0001

Abbreviations: sCr serum creatinine, ICU intensive care unit, LOS length of stay.

**Fig. 1:**
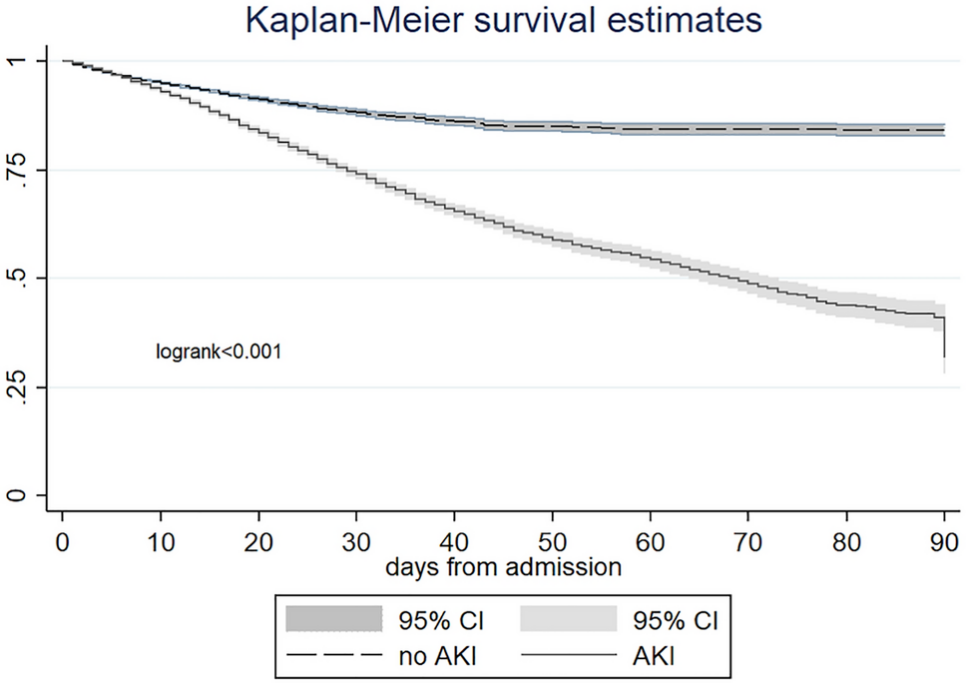
Kaplan–Meier curves of 90-day survival according to AKI development.

We also investigated mortality risk factors in the study cohort by using both univariate and multivariate Cox analyses. Univariate analysis revealed that age, male sex, ICU admission, sCr at admission, and the occurrence of AKI, were all significantly associated with mortality. In the multivariate analysis, the development of AKI remained a significant and independent predictor of mortality risk (HR 1.23; 95% CI 1.16–1.30) (Table 4). This finding was further confirmed by Kaplan–Meier survival analysis (Fig. 1).

**Table 4:** Univariate and multivariate Cox regression analyses for in-hospital mortality.

Risk factors	Univariate			Multivariate model		
	HR	95% CI	p	HR	95% CI	p
Sex (male)	1.06	1.01–1.12	0.013	1.11	1.06–1.17	< 0.0001
Age	1.05	1.05–1.05	< 0.0001	1.06	1.05–1.06	< 0.0001
Baseline sCr	1.18	1.17–1.2	< 0.0001	1.15	1.13 − 1.17	< 0.0001
Medical Ward	Ref					
ICU stay	3.16	2.96–3.38	< 0.0001	3.86	3.61–4.13	< 0.0001
AKI	1.9	1.8–2	< 0.0001	1.23	1.16–1.30	< 0.0001

Abbreviations: sCs: serum creatinine, ICU: intensive care unit.

### Correlation of outcomes with AKI KDIGO stages

**Fig. 2:**
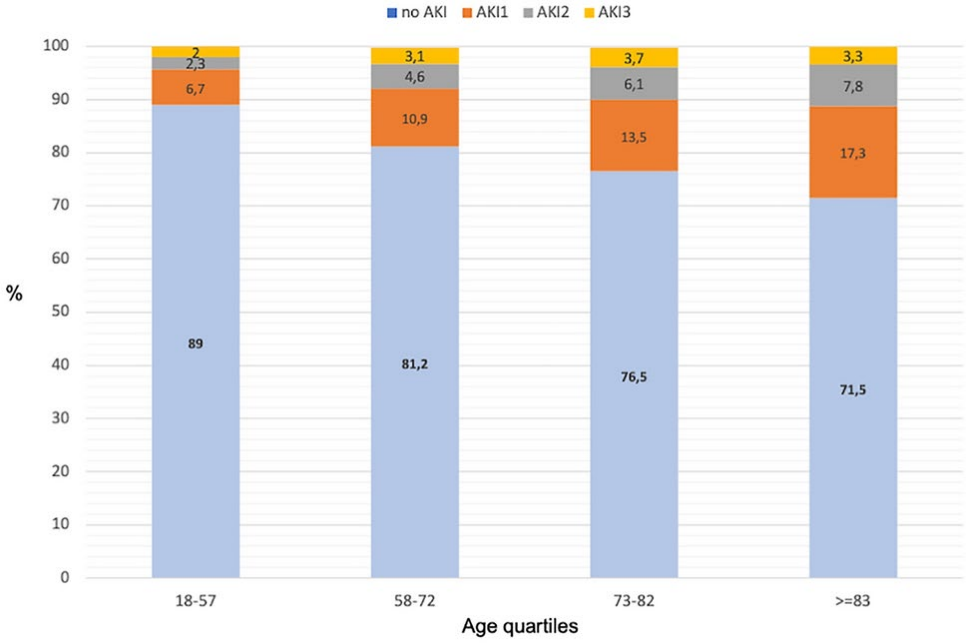
AKI stage distribution according to age quartiles.

The following step was to evaluate the impact of AKI severity on patients’ outcomes. We observed that 10,679 patients (59.5%) developed stage 1 AKI, 4611 (25.7%) stage 2, and 2656 (14%) stage 3, respectively (Table 5). Notably, the mean age was lower in the stage 3 group. As expected, age stratification revealed an increased incidence of AKI in patients in the older quartiles in accordance with the probable reduction of renal functional reserve (Fig. 2).

**Table 5:** Main clinical characteristics of patients according to AKI severity (KDIGO stages).

	All AKI	AKI- 1	AKI- 2	AKI- 3	P (Aki stages)
N,	17,946	10,679 (59.5)	4611 (25.7)	2656 (14)	
Age (years)	74.8 ± 14.7	74.8 ± 15	75.9 ± 14	73 ± 14.4	< 0.0001
Sex, male%	8598 (47.9)	5065 (47.4)	2181 (47.3)	1352 (50.9)	0.004
Comorbidities in HDF, %					
Diabetes	2009 (11.2)	1271 (11.9)	480 (10.4)	258 (9.7)	0.001
HF	2645 (14.7)	1582 (14.8)	733 (15.9)	330 (12.4)	< 0.0001
Sepsis	1913 (10.7)	787 (7.4)	677 (14.7)	449 (16.9)	< 0.0001
CKD	1704 (9)	878 (8.2)	451 (9.8)	375 (14.1)	< 0.0001
sCr at admission (mg/dl)	1.55 ± 1.53	1.27 ± 0.96	1.61 ± 1.29	2.6 ± 2.5	< 0.0001
eGFR at admission (ml/min)	81.6 ± 16	84 ± 15	79.5 ± 15.6	74.6 ± 18	< 0.0001
					AKI 3 (%)*
Hospital Ward					
Medicine	6951 (38.7)	4215 (39.4)	1673 (36.3)	1063 (40)	15.3
Surgery	3620 (20.2)	2371 (22.2)	824 (17.8)	425 (16)	11.7
ICU	1481 (8.2)	731 (6.8)	444 (9.7)	306 (11.5)	20.6
Emergency Medicine	5893 (32.8)	3361 (31.5)	1670 (36.2)	862 (32.4)	14.6

Abbreviations: HF heart failure, CKD chronic kidney disease, sCR serum creatinine, ICU intensive care unit, LOS length of stay.

AKI stages were also characterized by a different prevalence of comorbidities: when compared to stage 1 and stage 2, patients with stage 3 AKI showed a lower prevalence of diabetes and cardiac diseases and a concomitant higher prevalence of sepsis and pre-existing CKD. Moreover, a higher prevalence of stage 3 AKI was observed in patients admitted to ICU: conversely, patients hospitalized in low- and medium-intensity care settings showed a higher prevalence of stage 1 AKI (Table 5).

**Fig. 3:**
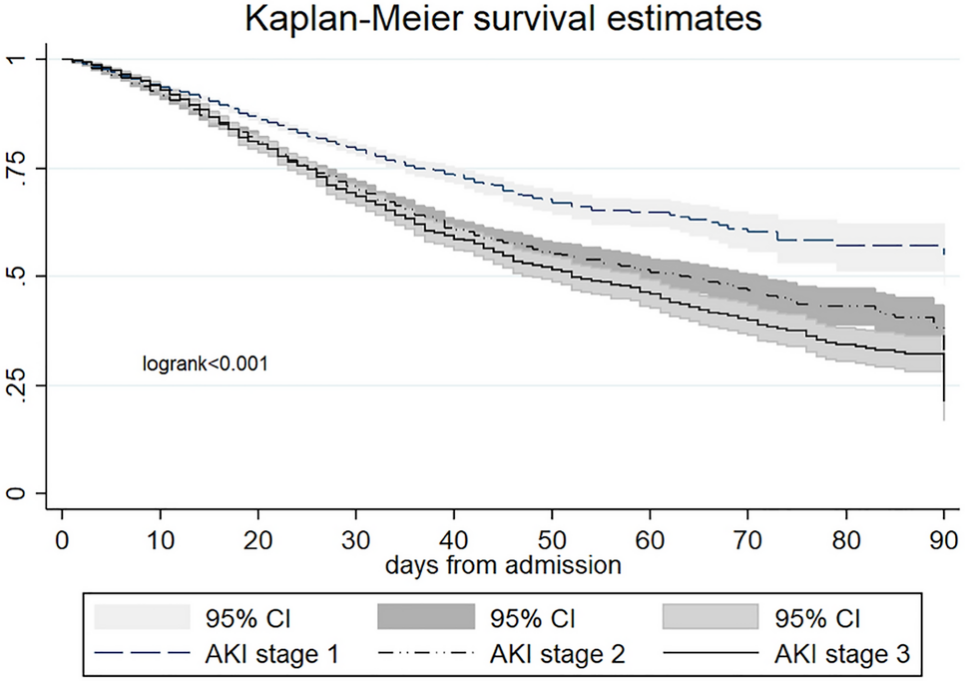
Kaplan–Meier curve of 90-day survival according to AKI stages.

The in-hospital mortality rate was significantly higher in stage 3 AKI group (Table 6): the 90-day Kaplan–Meier survival analysis confirmed a progressive increase in mortality in the most severe forms of AKI (Fig. 3). Furthermore, stage 3 AKI patients were more commonly admitted to ICU and had a longer LOS. The stage 3 group presented higher sCr levels at discharge and required more frequently protected assistance for the continuity of care (Table 6).

**Table 6:** Clinical outcomes according to the AKI stages.

	All AKI	AKI1	AKI2	AKI3	p (AKI stages)
N,	17,946	10,679 (59.5)	4611 (25.7)	2656 (14)	
In-hospital outcomes					
Mortality rate, %	3172 (17.7)	1245 (11.6)	1078 (23.4)	849 (32)	< 0.001
ICU admission, %	1481 (8.2)	731 (6.8)	444 (9.7)	306 (11.5)	< 0.001
LOS (days)	19.2 ± 16	16.3 ± 13.3	21.6 ± 17.9	27.1 ± 22.8	< 0.001
Discharge status- alive	14,774	9434	3533	1807	
Discharge at home, %	9382 (52.3)	6226 (66)	2091 (59)	1065 (59)	< 0.001
Protected discharge, %	5302 (30.05)	3208 (34)	1442 (40)	742 (41)	
sCr (mg/dl)	1.27 ± 1.09	1.15 ± 0.92	1.33 ± 1.09	1.67 ± 1.51	< 0.001
eGFR ml/min	84.86 ± 15.9	86.28 ± 0.15.3	83.36 ± 15.9	81.75 ± 17.7	< 0.001

Abbreviations: sCr serum creatinine, ICU intensive care unit, LOS length of stay.

### Outcomes in de-novo AKI vs. AKI on CKD

Pre-hospitalization sCr levels were available in 34,285 patients, constituting 39% of the total cohort.

These patients were analyzed to compare the clinical characteristics and outcomes of de novo AKI and AKI superimposed on pre-existing CKD.

Specifically, among this subgroup, 24,588 patients (71.8%) had no previous CKD, while 9697 patients (28.2%) presented with CKD before hospital admission, with a mean eGFR of 40.2 ± 14.1 ml/min.

De-novo AKI occurred in 17.3% (n. 4263) of patients without prior CKD, and AKI on CKD in 31.1% (n. 2795) of patients with pre-existing CKD.

AKI on CKD patients were older, prevalently males and with a higher prevalence of diabetes and HF (Table 7).

**Table 7:** Main clinical characteristics of AKI patients according to the presence of pre-existing CKD.

	AKI on CKD	De-novo AKI	p
N,	2795	4263	
Age (years)	79.0 ± 11.3	70.7 ± 14.7	< 0.0001
Gender M%	1436 (52.3)	2024 (47.5)	< 0.0001
Comorbidities, %			
Diabetes	430 (15.4)	434 (10.2)	< 0.0001
HF	611 (21.9)	478 (11.2)	< 0.0001
Sepsis	335 (12.0)	489 (11.5)	0.510
CKD	691 (24.7)	119 (2.8)	< 0.0001
sCr at admission (mg/dl)	2.45 ± 1.9	1.12 ± 0.88	< 0.0001
eGFR at admission (ml/min)	34.3 ± 28.8	74.8 ± 17.0	< 0.0001
AKI stage			< 0.0001
1	1562 (55.9)	2660 (62.4)	
2	798 (28.6)	1010 (23.7)	
3	435 (15.6)	593 (13.9)	

Abbreviations: HF heart failure, CKD chronic kidney disease, sCR serum creatinine, ICU intensive care unit, LOS length of stay.

**Fig. 4:**
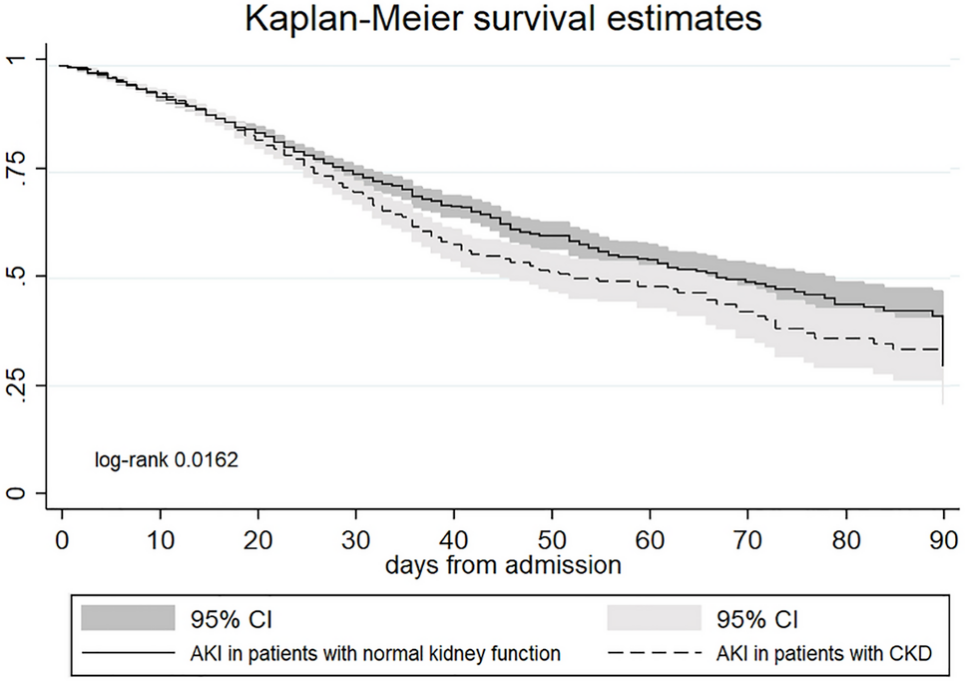
Kaplan–Meier curves of 90-day survival in accordance with the presence of pre-existing CKD. This analysis was restricted to patients with available pre-hospitalization eGFR (n = 34,285).

AKI on CKD patients presented higher sCr levels at admission and developed more severe AKI forms during hospitalization. The in-hospital mortality rate was significantly higher in AKI on CKD patients (Table 8): the 90-day Kaplan–Meier survival analysis confirmed these data (Fig. 4). Moreover, AKI on CKD patients were more frequently admitted to ICU, although the LOS did not differ in comparison to de-novo AKI patients. Last, AKI on CKD patients showed higher sCr levels at discharge.

**Table 8:** Clinical outcomes of all the population of hospitalized patients (2016–2029) according to the diagnosis of basal CKD based on previous serum creatinine levels.

	AKI on CKD	De-novo AKI	p
N,	2795	4263	
In-hospital outcomes			
Mortality rate, %	550 (19.7)	758 (17.8)	0.045
ICU admission, %	204 (7.3)	254 (5.9)	0.02
LOS (days)	19.2 ± 15.3	19.7 ± 17.8	0.232
Discharge status- alive	2245 (80.3)	3505 (82.2)	
Discharge at home, %	1624 (58.1)	2476 (58.1)	0.165
Protected discharge, %	621 (27.7)	1029 (29.4)	
sCr (mg/dl)	1.9 ± 1.41	0.99 ± 0.79	< 0.001

Abbreviations: sCr serum creatinine, ICU intensive care unit, LOS length of stay.

### Determinants and outcomes of Renal recovery or Persistent AKI

Finally, in the cohort of 14,774 AKI patients who survived hospitalization, we separately evaluated the incidence and determinants of renal recovery in comparison to the persistence of AKI at discharge. Overall, 12,175 (82.4%) patients experienced renal recovery, whereas 2599 (17.6%) had persistent AKI, still meeting the diagnostic criteria for KDIGO AKI according to serum creatinine levels at hospital discharge (Table 9).

**Table 9:** Main clinical characteristics of AKI patients hospitalized in the studied period (Jan 2016-Dec 2019), distinguished according to recovery status at the discharge.

	Recovery	Persistent AKI	P
N,	12,175 (82.4)	2599 (17.6)	
Age (years)	74.2 ± 15	73.8 ± 14.9	< 0.0001
Gender M%	5743 (47.2)	1222 (47)	0.9
Comorbidities, %			
Diabetes	1456 (12)	314 (12.1)	0.8
HF	1462 (12)	500 (19.3)	< 0.0001
Sepsis	965 (8)	189 (7)	0.2
CKD	1242 (10.2)	230 (8.9)	0.039
sCr at admission (mg/dl)	1.63 ± 1.6	1.16 ± 0.88	< 0.0001
eGFR at admission (ml/min)	59 ± 32	75 ± 31	< 0.0001
AKI stage			< 0.0001
1	8045 (66)	1388 (53.5)	
2	2754 (22.6)	777 (29.9)	
3	1376 (11.3)	434 (16.6)	

Abbreviations: CKD chronic kidney disease, HF hearth failure, sCr serum creatinine, ICU intensive care unit, AKI acute kidney injury.

There was no significant difference in sex distribution between these two groups. However, patients with persistent AKI were slightly but significantly younger, had a higher prevalence of HF, and a lower prevalence of CKD compared to those who recovered.

At admission, patients who recovered from AKI had significantly higher sCr levels compared to those with persistent AKI. Additionally, AKI severity differed between the groups, with a higher incidence of stages 2–3 AKI in the persistent AKI group (persistent severe AKI).

Multivariate logistic regression analysis indicated that the probability of renal recovery was significantly and positively associated with older age and a diagnosis of CKD (as reported in administrative records) (Table 10). Conversely, renal recovery was negatively associated with LOS, a history of HF, and ICU admission. Furthermore, clinical outcome analysis revealed that patients with persistent AKI were more frequently admitted to the ICU, had longer hospitalizations, and more often required a protected discharge post-hospitalization care (Table 11).

**Table 10:** Logistic models for the recovery after acute kidney injury in hospitalized patients.

Risk factors	Univariate			Multivariate model		
	OR	95% CI	p	OR	95% CI	p
Age	1.006	1.004–1.009	< 0.0001	1.007	1.004–1.01	< 0.001
Comorbidities						
Diabetes	0.98	0.87–1.12	0.84	1	0.87–1.14	0.9
HF	0.57	0.51–0.64	< 0.0001	0.52	0.46–0.59	< 0.0001
CKD	1.17	1.01–1.35	0.039	1.38	1.28–1.47	< 0.0001
AKI stage	0.71	0.67–0–76	< 0.0001	0.74	0.7–0.78	< 0.0001
ICU stay	0.75	0.65–0.86	< 0.0001	0.89	0.77–1.03	0.1
LOS (day)	0.99	0.98–0.99	< 0.0001	0.99	0.99–0.99	< 0.0001

Abbreviations: OR odds ratio, CI confidence interval, HF heart failure, CKD chronic kidney disease, sCr serum creatinine, ICU intensive care unit, LOS length of stay.

**Table 11:** Clinical outcomes of AKI patients hospitalized in the studied period (Jan 2016-Dec 2019) distinguished according to recovery status at the discharge.

	Recovery	Persistent AKI	p
N,	12,175 (82.4)	2599 (17.6)	
ICU admission, %	975 (8)	271 (10.4)	< 0.001
LOS (days)	18.5 ± 15.2	22.4 ± 22.1	< 0.001
Discharge at home, %	7786 (64)	1596 (61.5)	0.019
Protected discharge, %	4389 (36)	999 (38.5)	
sCr (mg/dl)	1.01 ± 0.69	1.56 ± 1.32	< 0.001

Abbreviations: sCr serum creatinine, ICU intensive care unit, LOS length of stay.

## Discussion

The aim of this study was to evaluate the incidence of AKI in patients admitted to two large University Hospitals in Italy between January 1, 2016, and December 31, 2019. We analyzed the clinical factors and outcomes associated with AKI development. The findings confirmed previous studies that showed AKI as an independent risk factor for increased in-hospital mortality, longer LOS, and progression to CKD. Using KDIGO criteria based solely on sCr values, we found an AKI incidence of 20.6% among hospitalized patients, which aligns with global AKI studies in our geographical region (South-West Europe)^13^. This supports the reliability of using sCr changes alone to define AKI. The most common form of AKI was stage 1, accounting for around 60% of cases.

We identified several risk factors for AKI, such as age, sex, and comorbidities. AKI patients were typically older and had a higher burden of comorbidities compared to non-AKI patients. In accordance with previous epidemiologic data including the 0by25 project of the International Society of Nephrology (ISN), AKI incidence was higher in patients admitted to the ICU, although about 20% of AKI cases also occurred in low- and medium-intensity care settings^12^. This highlights the need for further research on AKI in non-critically ill patients, an area currently underexplored in global AKI research.

The study also confirmed that AKI severity significantly impacts clinical outcomes^19^. AKI patients, especially those with severe forms, had worse conditions at discharge, with higher sCr levels and a greater need for protected follow-up. These patients may require long-term management, which has economic and social implications, and increases the risk of developing acute kidney disease (AKD) and progressing to CKD^20^. The relationship between AKI and CKD is complex and bidirectional, with CKD being a well-established risk factor for AKI^21^. We investigated this issue in our cohort, in which, in accordance with this selection strategy that excluded stages 4–5 CKD, only patients with mild forms of CKD were included.

Specifically, to ensure accuracy in estimating pre-hospitalization kidney function, we focused on comparing the characteristics and outcomes of de-novo AKI and AKI on CKD exclusively within the subgroup of patients with available pre-hospitalization eGFR.

In this cohort of 34,285 patients, our findings confirmed that individuals with pre-existing mild CKD exhibited a higher incidence of AKI.

These patients were older, had more comorbidities, and presented with elevated sCr at admission. They also developed more severe forms of AKI, which worsened clinical outcomes, highlighting the role of even mild CKD as an additional risk factor for AKI and its complications.

We also evaluated renal recovery in AKI survivors at hospital discharge. While most recovered their baseline kidney function, a significant portion (17.6%) still exhibited persistent AKI. Patients who recovered were generally older, had higher CKD prevalence, and had worse kidney function at admission but experienced less severe AKI during hospitalization. A plausible explanation is that many of these patients may have developed AKI in the community before hospitalization, which tends to have better outcomes as it is often caused by reversible factors^22,23^.

Interestingly, our findings partly contradict those of other studies, such as a recent review by Saraiva et al., which identified older age, diabetes, and CKD as risk factors for persistent AKI in non-renal transplant patients^24^. However, differences in the definitions of persistent AKI and patient populations likely explain these discrepancies. Indeed, while prior studies have largely focused on ICU patients^25,26^, our data pertains to a general hospital population, where ICU admissions represent a small minority.

Despite these differences, our results align with existing literature demonstrating that persistent AKI is associated to severe forms of the disease and worse outcomes, such as longer hospital stays, greater need for intensive care, and poorer discharge conditions^27,28^.

However, it should be emphasized that in this study, persistent AKI was defined based on creatinine levels at discharge, regardless of the length of hospitalization. Thus, we were unable to precisely determine the incidence of AKD, which current concepts on AKI trajectories recognize as a risk factor for subsequent progression to CKD^20^. Indeed, the formal definition of AKD (i.e., the persistence of kidney damage beyond 7 days from the onset of AKI) requires precise temporal data regarding the timing of AKI onset, which is lacking in our cohort^29^.

Another important factor to consider is the potential impact of AKI on renal function at discharge, highlighting the significance of renal functional reserve (RFR), defined as the kidney’s ability to increase GFR under physiological or pathological conditions^30^. Loss of RFR is an early step toward GFR decline and progression to CKD following an AKI episode^31^. This issue is likely to become increasingly important due to population growth, aging, and the rising incidence of comorbidities such as hypertension, diabetes, and obesity^32^.

Moreover, the worsening environmental conditions, such as global warming, pollution, and nephrotoxic drug use, will probably further worsen this scenario^33^.

The above-described epidemiological and clinical findings are confirmed at cellular and molecular level and nowadays AKI should be considered as a sort of accelerator of the aging processes within the kidney, leading to structural and functional changes, particularly in severe and persistent cases^34,35^. For this reason, these patients should receive specialized nephrological follow-up to optimize post-AKI care.

This study provided a comprehensive analysis of AKI intrahospital epidemiology, though we acknowledge certain limitations. The use of sCr-based AKI definitions may not fully capture all AKI cases, such as community-acquired AKI^6^.

Moreover, there are evidence suggesting that certain clinical conditions, such as sepsis and prolonged hospitalization—particularly in critical care settings—can affect serum creatinine production and its reliability as a marker of kidney function^36,37^.

However, in our study, we analyzed a large cohort of patients admitted to various hospital wards, where the occurrence of muscle loss is likely less pronounced compared to patients in the ICU. Nonetheless, the influence of muscle mass and nutritional status on serum creatinine is a well-known limitation of this marker. This limitation highlights the need for research and identification of novel renal biomarkers^38^.

Additionally, we lack information on AKI etiology, which could influence outcomes. The use of administrative codes to describe comorbidities may introduce biases, and we have no follow-up data, which are essential to understanding the full impact of in-hospital AKI on future health outcomes^39^.

Despite these limitations, our results are based on real-world data from two Italian university hospitals, making them valuable for further exploration. We addressed the unmet need in AKI management, particularly concerning the progression to CKD^40^. The recent UK ARID study demonstrated that CKD progression was significantly higher in hospitalized patients with severe AKI, supporting our findings^15^. Based on these considerations, the Italian Society of Nephrology’s Working Group on AKI and Extracorporeal Blood Purification Therapies recommends raising AKI awareness among clinicians, stakeholders, and policymakers. This consciousness derives from the analysis of epidemiological data coming from “real-life” evidence of AKI incidence not only focused on Intensive Care Units. Moreover, the application of the KDIGO criteria during the whole hospitalization will allow to identify patients with persistent and severe forms of AKI more prone to progress toward AKD and subsequently CKD^41^. Then, in these high-risk patients, establishing specialized post-AKI outpatient clinics, in collaboration with nephrologists and general practitioners, is crucial in warranting a proper follow-up. In this setting, given the advanced age of many AKI patients, incorporating telemedicine and digital health approaches could further enhance post-AKI care^42^. Moreover, creating biobanks to preserve plasma and urine samples for biomarker discovery would be a key step forward in improving AKI management and outcomes. Without these measures, the impact of AKI on mortality during hospitalization and CKD progression after discharge will likely worsen.

## Data Availability

The datasets used and/or analyzed during the current study are available from the corresponding author on reasonable request.

## References

[1] Kidney Disease: Improving Global Outcomes (KDIGO) Acute Kidney Injury Work Group. KDIGO clinical practice guideline for acute kidney injury. *Kidney Int Suppl (2011)* **2**, 4 (2012).

[2] Shiao, C.-C. et al. Long-term remote organ consequences following acute kidney injury. *Crit. Care* **19**, 438 (2015). 26707802 10.1186/s13054-015-1149-5 PMC4699348

[3] Coca, S. G., Singanamala, S. & Parikh, C. R. Chronic kidney disease after acute kidney injury: A systematic review and meta-analysis. *Kidney Int.* **81**, 442–448 (2012). 22113526 10.1038/ki.2011.379 PMC3788581

[4] Heung, M. & Chawla, L. S. Acute Kidney Injury: Gateway to Chronic Kidney Disease. *Nephron Clin. Pract.* **127**, 30–34 (2014). 25343817 10.1159/000363675

[5] Silver, S. A. & Chertow, G. M. The Economic Consequences of Acute Kidney Injury. *Nephron* **137**, 297–301 (2017). 28595193 10.1159/000475607 PMC5743773

[6] Esposito, P. et al. Recognition patterns of acute kidney injury in hospitalized patients. *Clin. Kidney J.*10.1093/ckj/sfae231 (2024). 39157067 10.1093/ckj/sfae231 PMC11328729

[7] Yokota, L. G. et al. Acute kidney injury in elderly patients: Narrative review on incidence, risk factors, and mortality. *Int. J. Nephrol. Renovasc. Dis.* **11**, 217–224 (2018). 30147352 10.2147/IJNRD.S170203 PMC6097506

[8] Ali, T. et al. Incidence and Outcomes in Acute Kidney Injury. *J. Am. Soc. Nephrol.* **18**, 1292–1298 (2007). 17314324 10.1681/ASN.2006070756

[9] Groeneveld, A. B. J., Tran, D. D., van der Meulen, J., Nauta, J. J. P. & Thijs, L. G. Acute Renal Failure in the Medical Intensive Care Unit: Predisposing, Complicating Factors and Outcome. *Nephron* **59**, 602–610 (1991). 1766500 10.1159/000186651

[10] Pickkers, P. et al. Acute kidney injury in the critically ill: an updated review on pathophysiology and management. *Intensive Care Med.* **47**, 835–850 (2021). 34213593 10.1007/s00134-021-06454-7 PMC8249842

[11] Mehta, R. L. et al. Spectrum of acute renal failure in the intensive care unit: The PICARD experience. *Kidney Int.* **66**, 1613–1621 (2004). 15458458 10.1111/j.1523-1755.2004.00927.x

[12] Mehta, R. L. et al. Recognition and management of acute kidney injury in the International Society of Nephrology 0by25 Global Snapshot: A multinational cross-sectional study. *The Lancet* **387**, 2017–2025 (2016). 10.1016/S0140-6736(16)30240-9 27086173

[13] Hoste, E. A. J. et al. Global epidemiology and outcomes of acute kidney injury. *Nat. Rev. Nephrol.* **14**, 607–625 (2018). 30135570 10.1038/s41581-018-0052-0

[14] Neyra, J. A. et al. Challenges in the Care of Patients with AKI Receiving Outpatient Dialysis: AKINow Recovery Workgroup Report. *Kidney360* **5**, 274–284 (2024). 38055734 10.34067/KID.0000000000000332 PMC10914193

[15] Horne, K. L. et al. A comprehensive description of kidney disease progression after acute kidney injury from a prospective, parallel-group cohort study. *Kidney Int.* **104**, 1185–1193 (2023). 37611867 10.1016/j.kint.2023.08.005

[16] Silver, S. A. et al. Association of an Acute Kidney Injury Follow-up Clinic With Patient Outcomes and Care Processes: A Cohort Study. *Am. J. Kidney Dis.* **81**, 554-563.e1 (2023). 36521779 10.1053/j.ajkd.2022.10.011

[17] International Classification of Diseases - 9th revision - Clinical Modification 2007. https://www.salute.gov.it/portale/documentazione/p6_2_2_1.jsp?lingua=italiano&id=2251.

[18] Esposito, P. et al. Changes of Acute Kidney Injury Epidemiology during the COVID-19 Pandemic: A Retrospective Cohort Study. *J. Clin. Med.* **11**, 3349 (2022). 35743418 10.3390/jcm11123349 PMC9225342

[19] Ikizler, T. A. et al. A prospective cohort study of acute kidney injury and kidney outcomes, cardiovascular events, and death. *Kidney Int.* **99**, 456–465 (2021). 32707221 10.1016/j.kint.2020.06.032 PMC7374148

[20] Kurzhagen, J. T., Dellepiane, S., Cantaluppi, V. & Rabb, H. AKI: An increasingly recognized risk factor for CKD development and progression. *J. Nephrol.* **33**, 1171–1187 (2020). 32651850 10.1007/s40620-020-00793-2

[21] Belayev, L. Y. & Palevsky, P. M. The link between acute kidney injury and chronic kidney disease. *Curr. Opin. Nephrol. Hypertens.* **23**, 149–154 (2014). 24384553 10.1097/01.mnh.0000441051.36783.f3 PMC4179244

[22] Wonnacott, A., Meran, S., Amphlett, B., Talabani, B. & Phillips, A. Epidemiology and Outcomes in Community-Acquired Versus Hospital-Acquired AKI. *Clin. J. Am. Soc. Nephrol.* **9**, 1007–1014 (2014). 24677557 10.2215/CJN.07920713 PMC4046725

[23] Mesropian, P. D. et al. Community-acquired acute kidney injury: A challenge and opportunity for primary care in kidney health. *Nephrology* **21**, 729–735 (2016). 26890822 10.1111/nep.12751

[24] Saraiva, I. E. et al. Risk Factors and Outcomes Associated With the Development of Persistent Acute Kidney Injury in Non-Renal Solid Organ Transplant Recipients: Systematic Review and Meta-Analysis. *Clin. Transplant.*10.1111/ctr.15444 (2024). 39190289 10.1111/ctr.15444 PMC11801782

[25] Kellum, J. A. Persistent Acute Kidney Injury*. *Crit. Care Med.* **43**, 1785–1786 (2015). 26181122 10.1097/CCM.0000000000001102 PMC4507291

[26] Samoni, S., De Rosa, S., Ronco, C. & Castellano, G. Update on persistent acute kidney injury in critical illnesses. *Clin. Kidney J.* **16**, 1813–1823 (2023). 37915904 10.1093/ckj/sfad107 PMC10616499

[27] Venkatachalam, M. A., Weinberg, J. M., Kriz, W. & Bidani, A. K. Failed Tubule Recovery, AKI-CKD Transition, and Kidney Disease Progression. *J. Am. Soc. Nephrol.* **26**, 1765–1776 (2015). 25810494 10.1681/ASN.2015010006 PMC4520181

[28] Perinel, S. et al. Transient and Persistent Acute Kidney Injury and the Risk of Hospital Mortality in Critically Ill Patients. *Crit. Care Med.* **43**, e269–e275 (2015). 25962084 10.1097/CCM.0000000000001077

[29] Molinari, L. et al. Distribution of Acute and Chronic Kidney Disease Across Clinical Phenotypes for Sepsis. *Chest* **166**, 480–490 (2024). 38462074 10.1016/j.chest.2024.03.006 PMC11443243

[30] Sharma, A., Mucino, M. J. & Ronco, C. Renal Functional Reserve and Renal Recovery after Acute Kidney Injury. *Nephron. Clin. Pract.* **127**, 94–100 (2014). 25343829 10.1159/000363721

[31] Husain-Syed, F. et al. Persistent decrease of renal functional reserve in patients after cardiac surgery-associated acute kidney injury despite clinical recovery. *Nephrol. Dial. Transplant.* **34**, 308–317 (2019). 30053231 10.1093/ndt/gfy227

[32] Chang-Panesso, M. Acute kidney injury and aging. *Pediatr. Nephrol.* **36**, 2997–3006 (2021). 33411069 10.1007/s00467-020-04849-0 PMC8260619

[33] Francis, A. et al. Chronic kidney disease and the global public health agenda: An international consensus. *Nat. Rev. Nephrol.* **20**, 473–485 (2024). 38570631 10.1038/s41581-024-00820-6

[34] Franzin, R. et al. Targeting Premature Renal Aging: From Molecular Mechanisms of Cellular Senescence to Senolytic Trials. *Front. Pharmacol.*10.3389/fphar.2021.630419 (2021). 33995028 10.3389/fphar.2021.630419 PMC8117359

[35] Agarwal, A. et al. Cellular and Molecular Mechanisms of AKI. *J. Am. Soc. Nephrol.* **27**, 1288–1299 (2016). 26860342 10.1681/ASN.2015070740 PMC4849836

[36] Prowle, J. R. et al. Serum creatinine changes associated with critical illness and detection of persistent renal dysfunction after AKI. *Clin. J. Am. Soc. Nephrol.* **9**, 1015–1023 (2014). 24742481 10.2215/CJN.11141113 PMC4046736

[37] Doi, K. et al. Reduced production of creatinine limits its use as marker of kidney injury in sepsis. *J. Am. Soc. Nephrol.* **20**, 1217–1221 (2009). 19389851 10.1681/ASN.2008060617 PMC2689892

[38] Strauß, C., Booke, H., Forni, L. & Zarbock, A. Biomarkers of acute kidney injury: From discovery to the future of clinical practice. *J. Clin. Anesth.* **95**, 111458 (2024). 38581927 10.1016/j.jclinane.2024.111458

[39] Esposito, P. et al. Renal Outcomes of Dialysis-Dependent Acute Kidney Injury in Noncritically Ill Patients: A Retrospective Study. *Blood Purif*10.1159/000517707 (2022). 34320502 10.1159/000517707

[40] Yeh, H.-C., Ting, I.-W., Huang, H.-C., Chiang, H.-Y. & Kuo, C.-C. Acute Kidney Injury in the Outpatient Setting Associates with Risk of End-Stage Renal Disease and Death in Patients with CKD. *Sci. Rep.* **9**, 17658 (2019). 31776433 10.1038/s41598-019-54227-6 PMC6881443

[41] Liu, K. D. et al. AKI!Now Initiative: Recommendations for Awareness, Recognition, and Management of AKI. *Clin. J. Am. Soc. Nephrol.* **15**, 1838–1847 (2020). 32317329 10.2215/CJN.15611219 PMC7769012

[42] Kashani, K. B. et al. Digital health and acute kidney injury: consensus report of the 27th Acute Disease Quality Initiative workgroup. *Nat. Rev. Nephrol.*10.1038/s41581-023-00744-7 (2023). 37580570 10.1038/s41581-023-00744-7 PMC11285755

